# Evaluation of glycemic control and related factors among outpatients with type 2 diabetes at Tikur Anbessa Specialized Hospital, Addis Ababa, Ethiopia: a cross-sectional study

**DOI:** 10.1186/s12902-022-00974-z

**Published:** 2022-03-07

**Authors:** Rodas Getachew Abera, Eyouel Shimeles Demesse, Wako Dedecha Boko

**Affiliations:** 1 Department of Medical Laboratory Sciences, College of Health Sciences, Addis Ababa University, Addis Ababa, Ethiopia; 2 Tikur Anbessa Specialized Hospital, College of Health Sciences, Addis Ababa University, Addis Ababa, Ethiopia

**Keywords:** Diabetes mellitus, Blood glucose control, Glycosylated hemoglobin, Tikur Anbessa, Ethiopia

## Abstract

**Background:**

The goals of glycemic management for patients with diabetes are to prevent or delay complications and optimize quality of life. However, in clinical practice, the recommended glycemic control target is difficult to achieve. Therefore, it is important to identify factors that influence the outcomes of glycemia to improve the quality of diabetic management.

The study aimed to evaluate the level and factors associated with glycemic control among type 2 diabetic outpatients at Tikur Anbessa Specialized Hospital, Addis Ababa, Ethiopia.

**Methods:**

A hospital-based cross-sectional study was conducted among systematically selected 325 patients with type 2 diabetes who attended diabetic clinics at Tikur Anbessa Specialized Hospital. Pretested, structured, and interviewer-administered questionnaires were used to collect sociodemographic and diabetes-related information from March 1 to May 30, 2021. HbA1c was used to assess glycemic control according to the HbA1c target of < 7% (‘good’ control) as recommended by the American Diabetes Association for non-pregnant adults. The HbA1c level in the range of 7–8% was defined as ‘inadequate’ control and ‘poor’ at levels > 8%. Data entry and analysis were performed using SPSS v26. Multivariate logistic regression analysis was used to identify determinants of glycemic control.

**Results:**

The median level of HbA1c of the participants was 8.4% (IQR 6.8–10.1). And approximately three-quarters (73.8%) of the patients had inadequate and poor glycemic control (HbA1c ≥ 7%). Older age (AOR: 2.46, 95% CI: 1.28–6.01), DM duration of > 10 years (AOR: 3.15, 95% CI: 2.22–6.54), insulin therapy (AOR: 3.07, 95% CI: 2.10–6.12), poor diet compliance (AOR: 1.97, 95% CI: 1.28–3.52) and failure to set goals for glycemic control (AOR: 3.42, 95% CI: 2.17–5.97) were factors associated with inadequate and poor glycemic control.

**Conclusions:**

The study revealed that a significant number of diabetic patients had inadequate and poor glycemic control levels. And this was associated with older age, longer duration of DM, insulin therapy, poor diet compliance, and failure to set control goals. This requires a focus on the associated factors identified and tailored management mechanisms to maintain good glycemic control.

**Supplementary Information:**

The online version contains supplementary material available at 10.1186/s12902-022-00974-z.

## Background

Diabetes mellitus (DM) is one of the main global health challenges of the twenty-first century. It is a metabolic disease characterized by chronic hyperglycemia caused by multiple etiologies, including defects in insulin secretion, action, or both [1]. Approximately 463 million adults (20–79 years) lived with diabetes worldwide in 2019 and about 4.2 million deaths were directly attributed to it [2, 3]. Type 2 DM is the most common form of DM, accounting for (~ 90% of patients), and the remaining 10% are type 1 diabetes or gestational diabetes. The prevalence of diabetes is rapidly increasing throughout the world, placing a severe economic burden on patients and society at large [4].

According to the International Diabetes Federation (IDF), an estimated 19.4 million adults aged 20–79 years lived with diabetes in the IDF Africa Region in 2019, representing a regional prevalence of 3.9%. The report also stated that Ethiopia is one of the most populous countries in Africa with the highest number of people with diabetes (~ 1.7 million) with a prevalence rate of 3.2% among adults [5].

Many previous studies have assessed glycemic control levels among patients with type 2 diabetes in Ethiopia and reported a wide range of values. Cross-sectional hospital-based studies conducted in cities/towns of Dire Dawa, Jimma, Gondar, Nekemete, Addis Ababa, Dessie, Debre Tabor, Mettu, and Debre Markos showed that 45.2%, 54.7%, 60.5%, 64.9%, 68.3%, 70.8%, 71.4%, 72.7%, 73.5% of the participants had poor glycemic control, respectively [6–14]. And this has been reported to be mainly associated with factors such as older age, longer duration of diabetes, insulin therapy, nonadherence to medications, poor diet adherence, and physical inactivity.

However, many, if not all, of these studies based their assessment on fasting blood glucose (FBG) levels. Very few have managed to measure the level of glycated hemoglobin (HbA1c) of participants. Using FBG over HbA1c, which is more accurate than the measurement of FBG to evaluate glycemic control, may not correctly represent the status of glycemic control of patients. FBG levels provide a short-term picture of control, while HbA1c is the most reliable indicator of long-term glycemic control as it accurately reflects an individual’s blood glucose levels during the preceding 2–3 months [15]. The nonuse of the HbA1c test in previous studies was described as a limitation and the need for further studies using the HbA1c test was implied in several studies [9, 11, 13, 16–19], by the respective investigators.

According to a previous study conducted in Ethiopia, only 18.6% of the patients had a history of the HbA1c test [20]. Other studies stated that none of the studied diabetic patients had a determination of HbA1c [21–24]. Furthermore, although most of the factors associated with glycemic control have been discussed, modifiable risk factors such as setting glycemic target goals and the general approach and participation of healthcare providers, particularly physicians, with patients that are crucial to achieving good glycemic control were not fully investigated in previous studies.

Therefore, to fill the existing gaps and provide objective and reliable information on the level of glycemic control for standard care provision for patients, the present study sought to evaluate the status of glycemic control and the underlying factors associated with inadequate & poor glycemic control among outpatients with T2DM using the National Glycohaemoglobin Standardization Program-certified (NGSP) and Diabetes Control & Complications Trial-standardized (DCCT) HbA1c technique in a tertiary healthcare setting in Ethiopia.

## Methods

### Study design, setting, and period

A hospital-based cross-sectional study was conducted among patients with T2DM attending the outpatient medical diabetic clinic of Tikur Anbessa Specialized Hospital (TASH), Addis Ababa, Ethiopia. The study was carried out for 3 months from March 1 to May 30, 2021. TASH is the largest and busiest public referral and teaching hospital in the country. The hospital has a bed capacity of 800 and offers diagnosis and treatment for more than 500,000 patients a year. The hospital’s endocrinology unit conducts two diabetes clinics a week and provides comprehensive diabetes care to approximately 800 to 1000 diabetic outpatients a month.

### Participant eligibility criteria

All patients with T2DM who attended the outpatient medical diabetic clinic of TASH and who had followed up in the clinic for at least one year were included in the study with their consent. On the other hand, patients receiving erythropoietin or blood transfusion and those with anemia or conditions that affect erythrocyte production were excluded from the study. Additionally, critically ill patients and pregnant women were also excluded from the study.

### Study variables

The dependent variable was the level of glycemic control (measured by the HbA1c test) and the independent variables were sociodemographic factors: age, sex, marital status, educational level, occupation, monthly income, residence, and access to healthcare. Clinical factors: duration of DM, family history of DM, body mass index (BMI), mode of therapy, presence of comorbidity, biochemical value. Behavioral factors: adherence to medications, adherence to diet, physical exercise, smoking, self-monitoring of blood glucose (SMBG), keeping up with follow-up visits, and setting glycemic target goals.

### Sample size and sampling method

The sample size was determined using a single population proportion formula considering a 59.4% proportion (p) of poor glycemic control as reported in an earlier study evaluated with HbA1c [7], with a confidence level of 95% and 5% marginal error (d).

$$n= \frac{(Z_{\propto}\!\left/_{2}\right.)^{2}\times p(1-p)}{{d}^{2}}$$

$$n= \frac{(1.96{)}^{2}\times 0.594*0.406}{({0.05)}^{2}}=371$$

The estimated average number of patients with T2DM expected to visit the diabetic clinic during the study period was *N* = 1434. Since the source population (N) had less than 10,000 respondents, the sample size was adjusted with a correction formula (*nf*):

$$nf= \frac{n}{1+ \frac{n}{N}}= \frac{371}{1+ \frac{371}{1434}}=295$$

Assuming 10% non-response rate: (0.1) * (295) **≈ 30.**

295 + 30 = **325.**

A systematic random sampling technique was used to select study participants at every k-th interval. The actual sampling fraction (k^th^) was determined by dividing the total number of the source population (1434) by the corrected sample size (295) ≈ 5. Therefore, every fifth patient was approached and invited to participate in the study until the required sample size was reached.

### Data collection procedure

Data were collected after the completion of the physician’s office visit session. After obtaining informed consent from each study participant, information about sociodemographic, behavioral, and clinical characteristics was recorded through face-to-face interviews using structured and pre-tested questionnaires. The patient’s medical records were also reviewed the same day after the interview to look for possible limiting factors that could interfere with the HbA1c test.

### Data collection tools

#### Questionnaire

The questionnaire was developed based on various similar previous studies [18, 25–28] and was further modified to include important variables from this study [see Additional file 1]. It was initially prepared in English, translated into Amharic, the local language, and retranslated into English again to ensure consistency. Data were collected in collaboration with outpatient department nurses & laboratory personnel under close supervision by the principal investigators. The World Health Organization (WHO) safety guidelines and protocols for COVID-19 were strictly followed at all times.

#### Laboratory examination

Laboratory investigations were performed for biochemical parameters: the level of glycated hemoglobin (HbA1c), fasting blood sugar (FBS), renal function test (RFT), and lipid profile [see Additional file 2]. 3 ml of freshly drawn venous blood was collected in an EDTA tube for the determination of HbA1c by a turbidimetric immunoinhibition method using the fully automated Beckman Coulter DxC 700 AU clinical chemistry analyzer. The technique has been certified by the NGSP [29] and is not affected by common hemoglobin variants (HbC, HbS, HbE, and HbD traits) and elevated fetal hemoglobin (HbF), minimizing inaccurate results for patients with these blood conditions [30]. 5 ml of venous blood samples were also drawn from study participants in serum separation tubes (SST), in which serum was used to measure the FBS, RFT, and lipid profiles of patients using the same analyzer.

### Data quality assurance

The quality of the data was ensured by properly designing the tool and the questionnaire was pre-tested in 5% of the randomly selected patients with T2DM at St. Paul’s Hospital Millennium Medical College before actual data collection, and some minor modifications were made accordingly. The principal investigators throughout the data collection process were in close contact and under close supervision. The completeness and consistency of the collected data were checked daily. Strict procedures were also implemented in the laboratory analysis of the analytes. A daily quality control test was performed for each analyte prior to sample analysis. Controls were also performed with each new lot of reagent and after specific maintenance or troubleshooting steps. A fasting-state blood sample was used for all analyte measurements. Samples were drawn into prelabeled barcoded SST and EDTA tubes, and quality & quantity were checked. Blood samples collected in test tubes were processed with as little delay as possible and brought to the laboratory for evaluation of biochemical parameters on the same day. The results obtained were properly coded and documented.

### Data analysis and interpretation

Data entry and analysis were performed using SPSS version 26. Descriptive statistics, including frequency, percentages, and median, were used to summarize baseline sociodemographic data from patients and evaluate the distribution of responses. Logistic regression analysis was conducted to look for any association between predictors and outcome variables. Factors with a P-value < 0.25 in the bivariate analysis were exported to the multivariate logistic regression analysis. Multivariate analysis using logistic regression was performed to control the effect of potential confounder variables and to identify independent predictors of inadequate & poor glycemic control. Consequently, statistically significant associations were determined based on the adjusted odds ratio (AOR) with its 95% CI and the *P*-value < 0.05.

Glycemic control was defined according to the HbA1c target of < 7% (‘good’ control) as recommended by the American Diabetes Association for non-pregnant adults. The HbA1c level in the range of 7–8% was defined as ‘inadequate’ control and ‘poor’ at levels greater than 8% [31].

### Operational definitions

#### Good glycemic control

Glycemic control was considered good if a patient had a HbA1c value < 7% [31].

#### Inadequate glycemic control

Glycemic control was considered inadequate if a patient had HbA1c values in the range of 7–8% [31].

#### Poor glycemic control

Glycemic control was considered poor if a patient had a HbA1c value > 8% [31].

#### Adherence to medication

if the study participant took all his/her antidiabetic medications in the last seven days [31].

#### Adherence to diet

If the study participant had followed the recommended diet for more than 3 days in the last seven days [31].

#### Adherence to exercise

If the study participant had followed the recommended level of exercise for more than 3 days in the last 7 days [31].

#### Diabetes complications

Harmful effects of diabetes, such as damage to the eyes, heart, blood vessels, nervous system, teeth and gums, feet, skin, or kidneys [31].

## Results

### Sociodemographic characteristics of the participants

A total of 325 patients with T2DM participated in the study. Among them, women comprised the majority of respondents, 186 (57.2%). The median age of the participants was 54 years (IQR 45–62). Two hundred and seventeen (66.8%) of the participants were married. One hundred twenty (36.9%) had completed their secondary education & one hundred six (32.6%) were either in college or had already earned their degrees. One hundred fifty-eight (48.6%) were government or private employees. More than half of the respondents (53.8%) were urban dwellers. One hundred eleven (34.2%) were making less than 1500 ETB a month. And a considerable number of study participants (60.6%) had access to hospital services free of charge (Table 1).

**Table 1:** Sociodemographic characteristics of study participants in TASH, Addis Ababa, Ethiopia, 2021

Variables	Category	Frequency (%)
Age, median (IQR)		54 years (45–62)
Age group (yrs.)	18-44	74 (22.8)
	45-54	92 (28.3)
	55-64	101 (31.1)
	≥ 65	58 (17.8)
Gender	Male	139 (42.8)
	Female	186 (57.2)
Marital status	Single	52 (16)
	Married	217 (66.8)
	Divorced	18 (5.5)
	Widow/er	38 (11.7)
Educational level	No formal education	37 (11.4)
	Primary ed. (grade 1-8)	62 (19.1)
	Secondary ed. (grade 9-12)	120 (36.9)
	College and above	106 (32.6)
Occupation	Unemployed	13 (4)
	Gov’t/Private Employee	158 (48.6)
	Self-employed	95 (29.2)
	Homemaker	24 (7.4)
	Retired/Pension	35 (10.8)
Monthly income (ETB)	< 1500	111 (34.2)
	1500-5000	130 (40)
	> 5000	84 (25.8)
Residence	Urban	175 (53.8)
	Rural	150 (46.2)
Healthcare access	Free	197 (60.6)
	Paid	128 (39.4)

*IQR* Interquartile Range, *ETB* Ethiopian Birr

### Clinical characteristics and disease management practices

The median duration of diabetes since diagnosis was 9 years (IQR 4–15). One hundred and fifty-three (47.1%) of the respondents implied that they had been living with diabetes for more than a decade. A family history of diabetes mellitus was recorded in at least one parent or child in 19.7% of the participants. Most of the respondents, 74.2% had a body mass index (18.5–24.9 kg/m^2^), normal weight, while 19.1% appeared to be overweight (25–29.9 kg/m^2^). Twelve (3.7%) were obese (≥ 30 kg/m^2^) and the remaining 3.1% fell within the underweight range (< 18.5 kg/m^2^). Comorbidities were present in almost 40% of the study participants. The most prevalent comorbidity was hypertension, which represented almost 20% of all comorbidities, followed by dyslipidemia (7.7%) and ischemic heart disease (3.1%). One hundred and fourteen (35.1%) respondents have already developed one or more diabetic complications. Diabetic neuropathy was the most common diabetes complication (14.2%) among the study participants. And 29 (9%) of the respondents were affected by multiple complications related to diabetes.

Only twelve (3.7%) of the participants were in a non-pharmacological mode of therapy. Most of the study participants (44.6%) used insulin, 93 (28.6%) were on oral hypoglycemic agents, and the rest 75 (23.1%) used combinations of insulin and oral hypoglycemic agents.

Over 50% of the participants had routine follow-up visits more than three times a year. About 70% adhered fully to their prescribed medications during the previous week before the study. A little more than half of the respondents 168 (51.7%) followed the recommended healthy eating plan adequately. And 138 (42.5%) participated in at least 30 min of physical activity for more than 3 days a week. 41.8% of the respondents have the means to self-monitor their blood glucose levels, own a glucometer, and the rest access nearby clinics or pharmacies. As few as eight (2.5%) participants were active smokers. And around 40% of the respondents indicated that they have set a glycemic target goal for management, which they strive to achieve (Table 2).

**Table 2:** Clinical characteristics and disease management practices of study participants in TASH, Addis Ababa, Ethiopia, 2021

Variables	Category	Frequency (%)
DM duration, median (IQR)		9 years (4–15)
Duration of DM	2-5 years	50 (15.4)
	6-10 years	122 (37.5)
	≥ 11 years	153 (47.1)
Family history of DM	Yes	64 (19.7)
	No	261 (80.3)
BMI (kg/m^2^)	Underweight (< 18.5)	10 (3.1)
	Normal weight (18.5–24.9)	241 (74.2)
	Overweight (25–29.9)	62 (19.1)
	Obese (≥ 30)	12 (3.7)
Mode of therapy	Oral hypoglycemic agents	93 (28.6)
	Insulin	145 (44.6)
	Combination of both	75 (23.1)
	Diet modification/Exercise alone	12 (3.7)
No. of follow-up visits	≤ 3 times/year	156 (48)
	> 3 times/year	169 (52)
Medication adherence (*n* = 313)	7 days/week (adequate)	228 (70.2)
	< 7 days/week (inadequate)	85 (26.2)
Diet adherence	> 3 days/week (adequate)	168 (51.7)
	0-3 days/week (inadequate)	157 (48.3)
Physical exercise	> 3 days/week (Adequate)	138 (42.5)
	0-3 days/week (Inadequate)	187 (57.5)
Access to SMBG	Yes	136 (41.8)
	No	189 (58.2)
Glycemic targets goal (HbA1c/FBS/RBS)	Yes	129 (39.7)
	No	196 (60.3)
Smoking status	Current smoker	8 (2.5)
	Ex-smoker (> 1 year)	16 (4.9)
	Nonsmoker	301 (92.6)
Co-morbidity	Present	129 (39.7)
	Absent	196 (60.3)
Type of co-morbidity(ies)	Hypertension	63 (19.4)
	Dyslipidemia	25 (7.7)
	Ischemic heart disease (IHD)	10 (3.1)
	Hypertension + IHD	7 (2.2)
	Others*	24 (7.4)
Complication	Present	114 (35.1)
	Absent	211 (64.9)
Type of complication(s)	Neuropathy	46 (14.2)
	Retinopathy	23 (7.1)
	Retinopathy + Neuropathy	21 (6.5)
	Retinopathy + Neuropathy + Nephropathy	8 (2.5)
	Cardiac complications	16 (4.9)

Others*—Asthma, Thyroid disorders & Obesity

*BMI* Body Mass Index, *SMBG* Self-monitoring of blood glucose, *HbA1c* Hemoglobin A1c, *FBS* Fasting blood sugar, *RBS* Random blood sugar

### Glycemic control & biochemical parameters

**Fig. 1:**
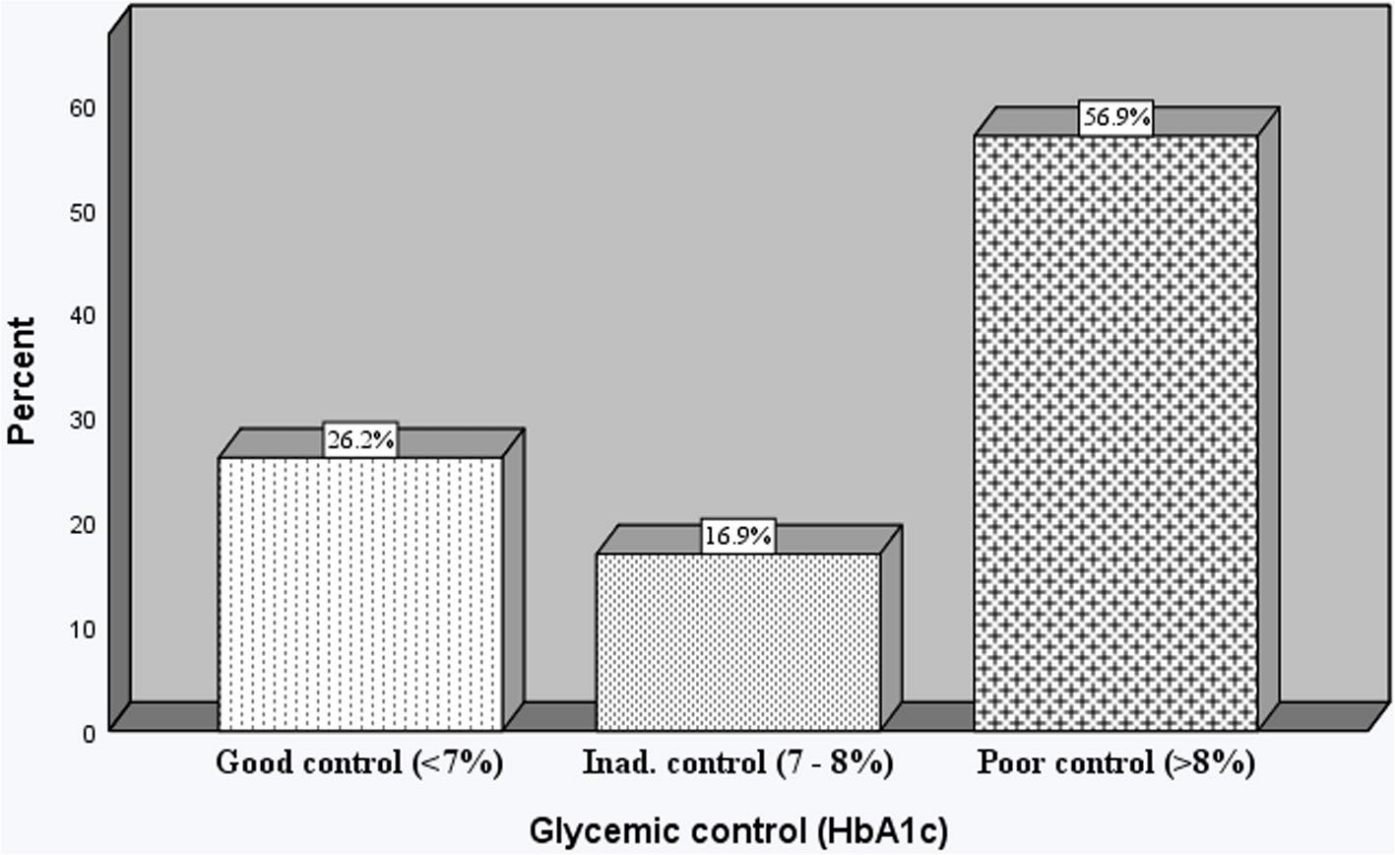
Level of glycemic control among patients with T2DM at TASH, Addis Ababa, Ethiopia, 2021

The median level of HbA1c of the study participants was found to be 8.4% (IQR 6.8–10.1). Good glycemic control was achieved only in 85 (26.2%) of the total respondents, according to the criteria of the American Diabetes Association of less than 7%. About 17% had inadequate control (7—8%). And more than half of the participants (56.9%) had poor glycemic control (> 8%) (Fig. 1). Fasting blood sugar level was greater than or equal to 130 mg/dL in 58.2% of the respondents, while approximately 80% of the respondents had a total cholesterol value below 200 mg/dL (Table 3).

**Table 3:** Biochemical parameters of the study participants in TASH, Addis Ababa, Ethiopia, 2021

Parameters	Category	Frequency (%)
HbA1c level, median (IQR)		8.4% (6.8–10.1)
FBS (mg/dL)	< 130	136 (41.8)
	≥ 130	189 (58.2)
Total cholesterol (mg/dL)	< 200	259 (79.7)
	≥ 200	66 (20.3)
Triglyceride (mg/dL)	< 150	210 (64.6)
	≥ 150	115 (35.4)
HDL-C (mg/dL)	M: ≥ 40, F: ≥ 50	171 (52.6)
	M: < 40, F: < 50	154 (47.4)
LDL-C (mg/dL)	< 130	211 (64.9)
	≥ 130	114 (35.1)
Urea (mg/dL)	10-50	308 (94.8)
	> 50	17 (5.2)
Creatinine (mg/dL)	0.5-1.2	297 (91.4)
	> 1.2	28 (8.6)

*HDL-C* High-density lipoprotein cholesterol, *LDL-C* Low-density lipoprotein cholesterol

### Factors associated with inadequate and poor glycemic control

In bivariate logistic regression: age, sex, duration since diagnosis of diabetes, body mass index, mode of therapy, adherence to diet, physical exercise, glycemic target goals, and comorbidities were associated with glycemic control and exported to the multivariate logistic regression model.

The results of the multivariate logistic regression analysis to identify factors associated with inadequate & poor glycemic control showed that older age, duration of DM of more than 10 years, insulin therapy, adherence to diet less than 3 days a week, and failure to set glycemic target goals were factors associated with inadequate & poor glycemic control (AOR: 2.46, 95% CI: 1.28–6.01, *P* = 0.03), (AOR: 3.15, 95% CI: 2.22–6.54, *P* = 0.016), (AOR: 3.07, 95% CI: 2.10–6.12, *P* = 0.022), (AOR: 1.97, 95% CI: 1.28–3.52, *P* = 0.002) and (AOR: 3.42, 95% CI: 2.17–5.97, *P* = 0.001), respectively.

The study showed that older age individuals with diabetes, specifically in the age category of (55–64) years, tend to have inadequate & poor control over their blood sugar levels compared to their younger counterparts. Patients with diabetes mellitus for more than ten years were found to be 3.15 times more likely to have inadequate & poor glycemic control than those with shorter durations. And the odds of having inadequate & poor glycemic control were found to be 3.07 times higher among patients on insulin therapy than among those on different treatment regimens. Furthermore, there was also a noticeable difference in adherence to a diet. Study participants who adhered to a diet for less than 3 days a week were approximately twice as likely to have inadequate & poor blood glucose control compared to those who adhered to the recommended healthy eating plan adequately. Finally, patients without established glycemic target goals were found to be 3.42 times more likely to have inadequate & poor glycemic control than those who established one (Table 4).

**Table 4:** Bivariate and multivariate logistic regression analyses of factors associated with glycemic control among patients with T2DM in TASH, Addis Ababa, Ethiopia, 2021

Variable	Category	Glycemic control (HbA1c)		Bivariate Analysis		Multivariate Analysis	
		Good (< 7%) *n* = 85	Inadequate-poor (≥ 7%) *n* = 240	COR (95% CI)	P-value	AOR (95% CI)	P-value
Age group (yrs.)	18-44	26	48	1	-	1	-
	45-54	26	66	0.74 (0.39–1.18)	0.042*	1.63 (0.66–4.18)	0.11
	55-64	16	85	1.90 (1.10–3.20)	0.002*	2.46 (1.28–6.01)	0.03*
	≥ 65	17	41	1.72 (1.02–3.06)	0.008*	1.97 (1.30–5.97)	0.04*
DM duration	≤ 10 years	72	100	1	-	1	-
	> 10 years	33	120	1.62 (1.13–3.43)	0.001*	3.15 (2.22–6.54)	0.016*
Mode of therapy	Oral hypoglycemic agents (OHA)	46	47	1	-	1	-
	Insulin	41	104	2.50 (1.30–4.50)	0.001*	3.07 (2.10–6.12)	0.022*
	OHA & Insulin	25	50	1.12 (0.24–3.72)	0.029*	2.36 (0.82–5.97	0.073
	Diet modification/Exercise alone	11	1	0.52 (0.38–2.88)	0.054	0.91 (0.52–3.53)	0.160
Diet adherence	> 3 days/week	80	88	1	-	1	-
	0-3 days/week	45	112	2.32 (1.35–3.99)	0.001*	1.97 (1.28–3.52)	0.002*
Glycemic target goal (HbA1c/FBS/RBS)	Yes	82	47	1	-	1	-
	No	49	147	2.61 (1.90–4.82)	0.001*	3.42 (2.17–5.97)	0.001*

^*^Statistically significant

*COR* Crude Odds Ratio, *AOR* Adjusted Odds Ratio, *CI* Confidence Interval

## Discussion

Measurement of HbA1c is considered an important diagnostic tool in monitoring diet control and therapeutic regimes during the treatment of diabetes. To our knowledge, this is the first study to evaluate the state of glycemic control using the NGSP certified and DCCT standardized HbA1c assay method and to highlight the determinants of inadequate & poor glycemic control in TASH. The study findings showed that almost three-quarters (73.8%) of the study participants had inadequate and poor glycemic control (HbA1c ≥ 7%). The prevalence of inadequate & poor glycemic control status was comparable to previous studies conducted in Saudi Arabia (74.9%) [28], Ghana (70%) [32], Uganda (73.52%) [33] and Northeast Ethiopia (70.8%) [11]. However, the magnitude of inadequate and poor glycemic control status in the current study was greater than previously reported in the United States (69%) [34], India (37.5%) [27], Tanzania (49.8%) [35], North West Ethiopia (60.5%) [36], Addis Ababa, Ethiopia (68.3%) [10] and East Ethiopia (45.2%) [6]. The higher proportion of poor glycemic control in the present study than previous studies conducted in parts of the country could be that patients seeking advanced treatment were referred to the Tikur Anbessa Specialized Hospital. However, the finding in the present study appeared to be lower than some studies reported in Nigeria (83.3%) [37], Kenya (81.6%) [38], and Addis Ababa, Ethiopia (80%) [24]. Although the studies mentioned above had the same study design and comparable sample sizes, there were variations in the reported numbers. The discrepancy between the present and previous studies conducted in sub-Saharan Africa, particularly Ethiopia, may have arisen mainly from differences in the types and methods of glucose measurement. Some researchers based their studies on FBS measurements and others on HbA1c. The other is the use of different assay methods, especially in the case of HbA1c determination, where the use of assays outside those certified by NGSP and standardized by DCCT gives falsely high or low readings in patients with hemoglobin variants and therefore compromises the comparability between HbA1c laboratories [29]. Other factors related to the clinical and sociodemographic characteristics of the study participants may also have contributed to the observed variations.

Older age, duration of DM of more than 10 years, insulin therapy, adherence to diet fewer than 3 days a week, and failure to set glycemic target goals were significantly associated with inadequate & poor glycemic control. The study showed that older age individuals with diabetes, specifically 55 years and older, tend to have inadequate & poor control over their blood glucose levels compared to their younger counterparts. This finding is consistent with results from previous studies [9, 37]. This could be explained in part due to the less stringent glycemic goal approach for older adult patients considering factors such as limited life expectancy, extensive comorbid conditions, and advanced microvascular or macrovascular complications, where the risk and burden outweigh the potential benefits of intensive control. Unlike these findings, older age was associated with good glycemic control in a study conducted in Palestine [39]. Elsous et al. associated older age with literacy, knowledgeability & experience and concluded that older individuals were more likely to have well-controlled glycemic levels, which was not the case in the present study. Some other studies also had similar results [32, 36].

Patients with a longer duration of DM (> 10 years) were found to be 3.15 times more likely to have inadequate & poor glycemic control than those with a shorter duration of the disease. And this finding is commonly shared among many related studies [14, 18, 34, 40]. Due to the chronic and progressive nature of diabetes, patients with a longer duration of the disease may eventually find it difficult to maintain good glycemic control. Impaired insulin secretion due to beta-cell dysfunction could explain this [41]. In line with previous studies [33, 39, 42], the odds of having inadequate & poor glycemic control were higher (3.07 times) among patients receiving insulin therapy than among those receiving different treatment regimens. Many patients with T2DM eventually need insulin therapy once the progression of the disease overcomes the effect of hypoglycemic agents [43]. This could be the reason for the increased prevalence of inadequate & poor glycemic control status among insulin users.

Non-compliance with diet recommendations was also associated with inadequate & poor glycemic control. Study participants who adhered to a diet for less than 3 days a week were approximately twice as likely to have inadequate and poor blood glucose control compared to those who adhered adequately to the recommended healthy eating plan, which was also highlighted in some other studies [13, 44]. Finally, patients who did not set target goals for glycemic management were 3.42 times more likely to have inadequate & poor glycemic control than those with an established plan. This could be due to a lack of awareness of target blood glucose levels to manage diabetes among diabetes patients, which has also been reflected in recently conducted studies [24, 45]. Furthermore, only 41.8% of the respondents were found to have the means to self-monitor their blood glucose levels, owning a glucometer, while the remaining 58.2% had to regularly visit nearby clinics or pharmacies to check their blood glucose levels. The numbers may be higher than reported in previous studies [44, 46, 47], but this could be because most of the participants in the present study resided in urban areas. Given the high prevalence of diabetes in the country, the access and distribution of SMBG devices are still far from sufficient.

## Strength and limitations

This study is the first to use the NGSP-certified/DCCT-standardized HbA1c method to assess the level of glycemic control and the factors associated with it among diabetic patients at Tikur Anbessa Specialized Hospital. However, the study adopted a cross-sectional study design and comprised a relatively smaller number of participants.

## Conclusions and recommendations

The results of this study showed that approximately three-quarters (73.8%) of the study participants had inadequate & poor glycemic control, which is far below the recommended standards. And this was found to be associated with factors such as older age, longer duration of DM, insulin therapy, poor diet compliance, and failure to set control goals. This calls for a focus on the associated factors identified and tailored management mechanisms to maintain good glycemic control.

Greater efforts must be made to address the factors associated with inadequate & poor glycemic control and optimize quality of life. Special attention should be paid to elderly patients, those with a longer duration of DM, and those on insulin therapy by increasing the frequency of hospital follow-up visits to assess and closely monitor their health status. Many previously conducted studies have also highlighted the factors mentioned above associated with poorly controlled diabetes. Therefore, increasing follow-up visits for patients with similar conditions could prove vital to achieving good glycemic control.

Various awareness initiatives should be held on diabetes care and ways of self-management of the disease. As type 2 diabetes can be progressive, patients should actively participate in the management process. They must be aware of the benefits of leading a healthy lifestyle and advised to strictly adhere to a healthy eating plan and engage in physical activity as often as possible. In addition, patients need the help of healthcare providers, especially physicians, to set a glycemic target to manage diabetes. Target goals should be individualized considering the duration of DM, age/life expectancy, comorbidities, advanced microvascular or macrovascular complications, unawareness of hypoglycemia, and the preference of the individual patient, as recommended by the American Diabetic Association. More stringent targets (HbA1c < 6.5%) are suggested for patients with DM of short duration, long life expectancy, and no significant cardiovascular disease (CVD) if they can be achieved safely without inducing significant hypoglycemia. Less stringent goals (HbA1c up to 8%) are recommended for patients with limited life expectancy, significant comorbidities, advanced microvascular or macrovascular complications, and those with frequent or severe episodes of hypoglycemia. The target goals should also be reevaluated over time to balance risks and benefits as patient factors change. In general, doctor office visits should be participatory and patients should actively participate in a shared decision-making process with clinicians on matters related to their health and care to better implement diabetes care goals.

In addition, to provide standard care to patients, objective and reliable information is needed on the magnitude of glycemic control. In this regard, clinically effective and reliable results should be obtained using HbA1c methods certified by the NGSP and standardized by the DCCT. Furthermore, a concerted effort is needed to increase the access and availability of tools for self-monitoring blood glucose levels. More research is warranted with a larger sample to ensure representativeness and investigate the association between glycemic control and different factors that progressively affect it.

## Supplementary Information

**Additional file 1.** (PDF 166 kb)

**Additional file 2.** (DOCX 35 kb)

## Data Availability

All relevant data are within the manuscript and its supporting files.

## Abbreviations

DCCT: Diabetes Control and Complications Trial
DM: Diabetes mellitus
FBG: Fasting blood glucose
HbA1c: Glycated hemoglobin
NGSP: National Glycohaemoglobin Standardization Program
T2DM: Type 2 diabetes mellitus
TASH: Tikur Anbessa Specialized Hospital
