# Supplementary material for: Evaluation of glycemic control and related factors among outpatients with type 2 diabetes at Tikur Anbessa Specialized Hospital, Addis Ababa, Ethiopia: a cross-sectional study
Source: BMC Endocr Disord. 2022 Mar 7;22:54. doi: 10.1186/s12902-022-00974-z (PMC8898656; doi:10.1186/s12902-022-00974-z)
Supplement: Supplementary file 1 — Additional file 1. (PDF 166 kb) [file 12902_2022_974_MOESM1_ESM.pdf]

Structured Questionnaires

Part I: Eligibility (Screening Questionnaire)

1. Do you suffer from any form of anemia?
   a. Yes ☐
   b. No ☐
2. Have you recently undergone a blood transfusion?
   a. Yes ☐
   b. No ☐
3. Are you alcoholic or do you regularly consume alcoholic drinks?
   a. Yes ☐
   b. No ☐
4. Are you pregnant? (For females only)
   a. Yes ☐
   b. No ☐
5. Has the consent been clearly explained and obtained?
   a. Yes ☐
   b. No ☐

NB: Stop the interview if the answer to questions 1, 2, 3, 4 is **"Yes"** or to 5 is **"No"**. And continue with the next participant, please.

Part II: Questionnaires on sociodemographic and healthcare access of the study participants

No.	Variables	Category
1.	Age ________ Years	18 - 44 ☐<br>45 - 54 ☐<br>55 - 64 ☐<br>≥ 65 ☐
2.	Sex	Male ☐<br>Female ☐
3.	Marital status	Single ☐<br>Married ☐<br>Divorced/separated ☐<br>Widow/er ☐
4.	Educational level	No formal education ☐<br>Primary ed. (grade 1 - 8) ☐<br>Secondary ed. (grade 9 - 12) ☐<br>College & above ☐
5.	Residence	Urban ☐<br>Rural ☐
6.	Occupation	Unemployed ☐<br>Gov't/Private Employee ☐<br>Self-employed ☐<br>Housewife ☐<br>Retired/Pension ☐
7.	Monthly Income (ETB) __________	< 1500 ☐<br>1500 - 5000 ☐<br>> 5000 ☐
8.	Healthcare access	Free ☐<br>Paid ☐

Part III: Questionnaires on clinical characteristics of the study participants

No.	Variables	Category
1.	DM duration _______Years	2 - 5 years ☐<br>6 - 10 years ☐<br>≥ 11 years ☐
2.	Family History of DM	Yes ☐<br>No ☐
3.	BMI (kg/m^2)<br>(Write your answers)	Weight (kg) _______<br>Height (m) ________
4.	Mode of therapy	Oral hypoglycemic agents ☐<br>Insulin ☐<br>Combination of both ☐<br>Diet modification/Exercise alone ☐
5.	How many times per year do you show up to your follow-up clinic visit appointments? ________ times/yr.	≤ 3 times/year ☐<br>> 3 times/year ☐
6.	Do you take your prescribed medications at the right time & dosage without ever forgetting?	Yes, 7 days/week (Adequate) ☐<br>No, < 7 days/ week (Inadequate) ☐
7.	Do you strictly adhere to your recommended diet program? (Avoiding fatty foods & consuming more vegetables & fruits).	Yes, > 3 days/week (adequate) ☐<br>No, 0 - 3 days/week (in adequate) ☐
8.	How many times a week do you engage in physical activities?	> 3 days/week (Adequate) ☐<br>0 - 3 days/week (Inadequate) ☐
9.	Do you've access to a device to self-monitor your blood glucose level (SMBG)?	1. Yes (own glucometer) ☐<br>Yes (access nearby clinic/pharmacy ☐<br>2. No ☐
10.	Have you set glycemic target goals for management? (HbA1c/FBS/RBS)	Yes ☐<br>No ☐

11.	Smoking status	Current smoker ☐<br>Ex-smoker (>1 year) ☐<br>Non-smoker ☐
12.	Presence of co-morbidity?	Yes ☐<br>No ☐
13.	Type of co-morbidity(ies)<br>(Can tick more than once)	Hypertension ☐<br>Dyslipidemia ☐<br>Obesity ☐<br>Ischemic Heart Disease ☐<br>Peripheral Vascular disease ☐<br>Others, Specify ____________________________
14.	Presence of complication?	Yes ☐<br>No ☐
15.	Type of complication(s)<br>(Can tick more than once)	Retinopathy ☐<br>Neuropathy ☐<br>Nephropathy ☐<br>Coronary Heart disease ☐<br>Diabetic Foot Ulcer ☐<br>Others, Specify ____________________________
