# Supplementary material for: Evaluation of glycemic control and related factors among outpatients with type 2 diabetes at Tikur Anbessa Specialized Hospital, Addis Ababa, Ethiopia: a cross-sectional study
Source: BMC Endocr Disord. 2022 Mar 7;22:54. doi: 10.1186/s12902-022-00974-z (PMC8898656; doi:10.1186/s12902-022-00974-z)
Supplement: Supplementary file 2 — Additional file 2. (DOCX 35 kb) [file 12902_2022_974_MOESM2_ESM.docx]

**Clinical laboratory values of study participants**

| **No.** | **ID** | **Age**  **(yrs.)** | **Sex** | **A_1c_**  **(%)** | **FBS**  **(mg/dL)** | **Urea (mg/dL)** | **Cr.**  **(mg/dL)** | **TC (mg/dL)** | **TG (mg/dL)** | **HDL (mg/dL)** | **LDL (mg/dL)** |
| --- | --- | --- | --- | --- | --- | --- | --- | --- | --- | --- | --- |
|  | 35927 | 47 | F | 11 | 244 | 22 | 0.6 | 220 | 244 | 47 | 149.2 |
|  | 20951 | 49 | M | 6.5 | 175 | 28 | 0.9 | 112 | 53 | 60 | 55 |
|  | 69410 | 48 | M | 5.9 | 115 | 106 | 5.7 | 132 | 48 | 61 | 74.2 |
|  | 102338 | 65 | F | 6.8 | 113 | 26 | 0.5 | 152 | 83 | 35 | 107 |
|  | 589 | 59 | F | 11.7 | 215 | 13 | 0.7 | 129 | 120 | 40 | 82 |
|  | 30134 | 49 | M | 10.3 | 140 | 19 | 0.9 | 147 | 152 | 24 | 108.4 |
|  | 11226 | 35 | F | 6.5 | 99 | 29 | 0.8 | 205 | 201 | 56 | 138.6 |
|  | 7802 | 58 | F | 5.5 | 76 | 18 | 0.9 | 180 | 154 | 43 | 120 |
|  | 30101 | 40 | M | 10.6 | 208 | 27 | 1.0 | 133 | 140 | 31 | 93.8 |
|  | 101598 | 56 | M | 7.9 | 159 | 16 | 0.8 | 187 | 155 | 50 | 131.1 |
|  | 35741 | 41 | F | 7.4 | 136 | 9 | 0.8 | 203 | 123 | 52 | 148 |
|  | 13639 | 68 | F | 6.8 | 155 | 17 | 0.5 | 171 | 118 | 45 | 118 |
|  | 22007 | 82 | F | 6 | 115 | 21 | 0.5 | 194 | 132 | 67 | 127.8 |
|  | 42645 | 72 | F | 6.6 | 127 | 11 | 0.8 | 175 | 152 | 43 | 131.6 |
|  | 3291 | 45 | M | 5 | 98 | 23 | 0.6 | 104 | 93 | 40 | 71.2 |
|  | 48104 | 58 | F | 6 | 119 | 22 | 0.8 | 229 | 253 | 59 | 165.4 |
|  | 34922 | 72 | F | 10.4 | 236 | 17 | 0.5 | 92 | 44 | 38 | 58.9 |
|  | 35238 | 38 | F | 6.4 | 143 | 21 | 0.7 | 194 | 223 | 37 | 125 |
|  | 39959 | 52 | F | 11 | 262 | 22 | 0.6 | 168 | 395 | 32 | 79 |
|  | 36973 | 47 | F | 7.1 | 120 | 21 | 0.6 | 234 | 268 | 38 | 154 |
|  | 639 | 73 | F | 7.1 | 110 | 50 | 1.3 | 257 | 151 | 39 | 190 |
|  | 38619 | 47 | M | >15 | 359 | 26 | 0.9 | 162 | 117 | 25 | 116 |
|  | 101896 | 30 | M | 10.3 | 79 | 33 | 0.9 | 186 | 72 | 51 | 129.2 |
|  | 37341 | 37 | M | 5.9 | 102 | 23 | 0.9 | 158 | 123 | 28 | 108 |
|  | 21077 | 41 | F | 5.7 | 115 | 14 | 0.7 | 142 | 96 | 33 | 99 |
|  | 101933 | 59 | M | >15 | 327 | 15 | 0.8 | 208 | 177 | 54 | 130 |
|  | 18729 | 58 | F | 6.8 | 124 | 21 | 0.5 | 213 | 114 | 56 | 156.4 |
|  | 35021 | 72 | M | 9 | 139 | 17 | 0.8 | 77 | 63 | 30 | 467 |
|  | 101879 | 46 | M | 5.9 | 101 | 28 | 0.9 | 252 | 539 | 36 | 137 |
|  | 80796 | 41 | M | >15 | 179 | 13 | 0.7 | 142 | 145 | 40 | 108 |
|  | 49764 | 42 | F | 9.8 | 197 | 26 | 0.6 | 152 | 146 | 46 | 108.6 |
|  | 31806 | 77 | F | >15 | 242 | 18 | 0.7 | 167 | 193 | 44 | 121 |
|  | 15463 | 45 | F | 8.5 | 211 | 14 | 0.7 | 179 | 103 | 47 | 180.7 |
|  | 1464 | 63 | M | 7.9 | 193 | 69 | 1.8 | 86 | 75 | 33 | 55 |
|  | 65506 | 56 | F | 11.3 | 124 | 20 | 0.5 | 212 | 114 | 56 | 155 |
|  | 23632 | 57 | F | >15 | 374 | 29 | 0.9 | 181 | 529 | 36 | 122 |
|  | 19953 | 77 | M | 7.6 | 119 | 32 | 0.9 | 195 | 126 | 50 | 135 |
|  | 34137 | 65 | M | 11.1 | 191 | 24 | 0.9 | 102 | 185 | 33 | 71 |
|  | 32557 | 60 | F | 8.4 | 229 | 37 | 0.8 | 168 | 121 | 52 | 115 |
|  | 28391 | 50 | F | 8 | 230 | 31 | 0.6 | 147 | 90 | 54 | 104 |
|  | 14146 | 66 | F | 8 | 128 | 25 | 1.3 | 111 | 88 | 35 | 79 |
|  | 12805 | 63 | F | 9.0 | 106 | 23 | 0.7 | 132 | 203 | 36 | 88 |
|  | 24774 | 57 | M | 10.2 | 70 | 27 | 0.7 | 152 | 87 | 50 | 91 |
|  | 22227 | 62 | F | 7.8 | 141 | 17 | 0.9 | 124 | 243 | 46 | 82.5 |
|  | 39666 | 69 | M | 9.9 | 253 | 20 | 0.9 | 202 | 124 | 48 | 88 |
|  | 26358 | 48 | F | 7.9 | 159 | 15 | 0.6 | 167 | 195 | 30 | 125.8 |
|  | 91622 | 50 | F | 10.7 | 146 | 28 | 0.5 | 110 | 56 | 49 | 68 |
|  | 70633 | 56 | M | 9.2 | 161 | 15 | 1.2 | 93 | 70 | 44 | 57 |
|  | 44837 | 42 | F | 6.2 | 102 | 18 | 0.7 | 159 | 186 | 60 | 104 |
|  | 94742 | 35 | M | 10.6 | 302 | 17 | 0.7 | 152 | 115 | 45 | 109 |
|  | 4267 | 63 | F | 8.7 | 304 | 32 | 1.5 | 132 | 188 | 32 | 99 |
|  | 44426 | 67 | M | 9.3 | 206 | 97 | 2.9 | 137 | 184 | 35 | 102.5 |
|  | 5077 | 51 | M | 10.7 | 392 | 30 | 0.9 | 164 | 98 | 75 | 98 |
|  | 53203 | 53 | M | 8.6 | 198 | 26 | 0.9 | 115 | 200 | 37 | 80.9 |
|  | 1753 | 48 | M | 7.8 | 130 | 12 | 0.9 | 91 | 56 | 51 | 48 |
|  | 96193 | 57 | F | >15 | 243 | 46 | 0.9 | 227 | 98 | 68 | 150 |
|  | 18449 | 57 | M | 8.2 | 182 | 46 | 1.3 | 175 | 103 | 54 | 118 |
|  | 35425 | 48 | F | 8.3 | 214 | 19 | 0.6 | 190 | 202 | 49 | 145 |
|  | 14064 | 61 | F | 8 | 187 | 16 | 0.6 | 234 | 121 | 63 | 160 |
|  | 6173 | 62 | M | 6.3 | 119 | 29 | 1.4 | 152 | 78 | 54 | 102 |
|  | 57126 | 63 | M | 9.1 | 327 | 43 | 1.0 | 208 | 226 | 31 | 167 |
|  | 37418 | 64 | F | >15 | 368 | 21 | 0.6 | 186 | 91 | 48 | 134 |
|  | 9406 | 61 | M | 9.0 | 202 | 17 | 0.7 | 185 | 245 | 40 | 148 |
|  | 26163 | 60 | F | 7.2 | 110 | 26 | 0.5 | 178 | 79 | 71 | 114 |
|  | 5620 | 50 | M | 7 | 127 | 16 | 0.6 | 226 | 609 | 36 | 149 |
|  | 10442 | 39 | F | 10.1 | 254 | 15 | 0.6 | 213 | 126 | 44 | 169 |
|  | 101110 | 45 | F | 5.8 | 91 | 9 | 0.6 | 160 | 73 | 55 | 113 |
|  | 4828 | 63 | M | 10.4 | 158 | 31 | 0.8 | 114 | 79 | 38 | 80 |
|  | 30533 | 62 | F | 10.8 | 167 | 12 | 0.4 | 154 | 113 | 48 | 112 |
|  | 66586 | 38 | M | 9.1 | 95 | 22 | 0.8 | 113 | 127 | 38 | 78 |
|  | 101323 | 51 | M | 7.7 | 296 | 12 | 0.8 | 169 | 209 | 42 | 130 |
|  | 93490 | 60 | F | 10.9 | 175 | 39 | 0.5 | 220 | 109 | 53 | 164 |
|  | 86333 | 65 | F | 11.1 | 202 | 12 | 0.5 | 93 | 135 | 30 | 66 |
|  | 9406 | 61 | M | 8.6 | 202 | 17 | 0.7 | 185 | 245 | 40 | 148 |
|  | 26163 | 60 | F | 8.1 | 110 | 26 | 0.5 | 178 | 79 | 71 | 114 |
|  | 6470 | 57 | F | 10 | 76 | 38 | 1.2 | 102 | 40 | 36 | 69 |
|  | 90546 | 27 | F | 6 | 97 | 14 | 0.4 | 137 | 73 | 45 | 100 |
|  | 101080 | 60 | F | 9.3 | 178 | 12 | 0.5 | 162 | 83 | 51 | 118 |
|  | 12469 | 64 | F | 7.6 | 151 | 14 | 0.4 | 181 | 110 | 50 | 140 |
|  | 15492 | 52 | F | 7.4 | 172 | 17 | 0.4 | 154 | 121 | 52 | 106 |
|  | 36436 | 62 | M | 7 | 121 | 21 | 0.9 | 114 | 79 | 43 | 77 |
|  | 37185 | 37 | F | 6.3 | 133 | 18 | 0.5 | 240 | 211 | 45 | 195 |
|  | 13015 | 48 | M | 6.9 | 150 | 36 | 1.4 | 187 | 184 | 42 | 115 |
|  | 29238 | 50 | F | 9.4 | 140 | 15 | 0.6 | 114 | 98 | 44 | 76 |
|  | 24238 | 61 | F | 8.6 | 180 | 17 | 1.1 | 126 | 198 | 45 | 73 |
|  | 39605 | 43 | M | 6.9 | 112 | 49 | 1.1 | 177 | 133 | 45 | 128 |
|  | 38334 | 59 | F | 7.0 | 141 | 22 | 0.6 | 184 | 142 | 55 | 133 |
|  | 20755 | 59 | F | 7.9 | 109 | 12 | 0.5 | 123 | 109 | 55 | 81 |
|  | 72596 | 40 | F | >15 | 305 | 29 | 0.7 | 227 | 118 | 46 | 168 |
|  | 3639 | 59 | M | >15 | 239 | 39 | 1.3 | 127 | 114 | 41 | 89 |
|  | 31318 | 54 | F | 9 | 233 | 25 | 0.8 | 241 | 186 | 49 | 170 |
|  | 6708 | 48 | F | 11.6 | 92 | 17 | 0.8 | 137 | 134 | 41 | 102 |
|  | 29303 | 44 | M | 7.8 | 152 | 22 | 0.7 | 206 | 199 | 47 | 163 |
|  | 47109 | 78 | M | 5.5 | 92 | 27 | 0.7 | 106 | 60 | 42 | 66 |
|  | 26328 | 45 | F | 8.4 | 210 | 27 | 0.8 | 150 | 105 | 46 | 114 |
|  | 12241 | 44 | M | 7.4 | 132 | 14 | 0.7 | 116 | 177 | 42 | 81 |
|  | 26155 | 70 | M | 10.8 | 244 | 40 | 0.9 | 93 | 71 | 33 | 72 |
|  | 37710 | 60 | F | 6.8 | 93 | 13 | 0.9 | 151 | 100 | 50 | 95 |
|  | 91622 | 50 | F | 9.7 | 146 | 28 | 0.5 | 110 | 56 | 49 | 68 |
|  | 37196 | 72 | M | 88 | 165 | 30 | 0.9 | 100 | 108 | 29 | 76 |
|  | 61415 | 49 | M | 6.4 | 154 | 19 | 1.0 | 205 | 98 | 39 | 149 |
|  | 70933 | 47 | F | 6.9 | 129 | 18 | 0.7 | 153 | 169 | 38 | 123 |
|  | 49281 | 32 | F | 10.6 | 282 | 10 | 0.4 | 145 | 69 | 36 | 123 |
|  | 31286 | 28 | M | >15 | 220 | 24 | 0.8 | 142 | 56 | 51 | 110 |
|  | 12794 | 49 | F | 5.4 | 125 | 12 | 0.7 | 108 | 107 | 47 | 66 |
|  | 103769 | 50 | F | 8.2 | 88 | 23 | 0.7 | 215 | 128 | 67 | 160 |
|  | 72596 | 46 | F | >15 | 305 | 29 | 0.7 | 227 | 118 | 46 | 168 |
|  | 14688 | 72 | F | 6.1 | 102 | 20 | 0.5 | 129 | 85 | 53 | 92 |
|  | 11198 | 40 | F | 11.5 | 443 | 29 | 1.0 | 214 | 237 | 41 | 183 |
|  | 99821 | 48 | M | 9 | 196 | 30 | 0.9 | 90 | 137 | 29 | 55 |
|  | 27425 | 62 | M | 8.4 | 123 | 31 | 1.0 | 155 | 107 | 55 | 113 |
|  | 31416 | 62 | F | 8.5 | 194 | 23 | 0.6 | 134 | 110 | 52 | 98 |
|  | 102284 | 55 | F | 7.6 | 125 | 29 | 0.6 | 262 | 181 | 36 | 209 |
|  | 30519 | 66 | F | 8.8 | 309 | 17 | 1.0 | 132 | 118 | 51 | 94 |
|  | 20722 | 67 | F | 6.7 | 100 | 43 | 1.1 | 202 | 223 | 40 | 170 |
|  | 12962 | 73 | F | 7.7 | 214 | 37 | 1.2 | 131 | 90 | 45 | 76 |
|  | 5067 | 31 | F | 6.1 | 81 | 18 | 0.6 | 153 | 62 | 49 | 124 |
|  | 75294 | 61 | M | 5.8 | 84 | 42 | 0.7 | 215 | 97 | 46 | 142 |
|  | 104357 | 45 | F | >15 | 433 | 11 | 0.7 | 167 | 202 | 32 | 133 |
|  | 16395 | 81 | M | 10.1 | 265 | 25 | 1.2 | 171 | 119 | 43 | 131 |
|  | 65969 | 42 | M | 10.9 | 126 | 27 | 1.0 | 142 | 136 | 45 | 97 |
|  | 84541 | 41 | F | 6.5 | 129 | 23 | 0.6 | 152 | 175 | 47 | 104 |
|  | 98522 | 77 | M | 6.4 | 129 | 22 | 0.8 | 223 | 230 | 58 | 167 |
|  | 34310 | 30 | M | 7.3 | 83 | 11 | 0.6 | 136 | 61 | 44 | 99 |
|  | 98522 | 77 | M | 6.4 | 129 | 22 | 0.8 | 223 | 230 | 58 | 167 |
|  | 4267 | 63 | F | 9.2 | 130 | 32 | 1.5 | 132 | 188 | 32 | 99 |
|  | 24016 | 73 | F | 6.7 | 105 | 15 | 0.5 | 192 | 84 | 56 | 137 |
|  | 27067 | 62 | M | >15 | 244 | 37 | 1.1 | 122 | 151 | 32 | 94 |
|  | 92381 | 65 | F | 4.8 | 93 | 84 | 1.9 | 107 | 83 | 58 | 53 |
|  | 102671 | 53 | F | 7.3 | 163 | 19 | 0.6 | 159 | 69 | 41 | 130 |
|  | 5235 | 48 | M | >15 | 303 | 24 | 0.9 | 143 | 63 | 53 | 102 |
|  | 53127 | 39 | F | 8.4 | 175 | 26 | 0.8 | 180 | 216 | 44 | 109 |
|  | 6504 | 36 | M | 10.4 | 75 | 16 | 0.9 | 168 | 74 | 40 | 127 |
|  | 6708 | 48 | F | 10.9 | 92 | 17 | 0.8 | 137 | 134 | 41 | 102 |
|  | 53238 | 42 | F | 7.1 | 181 | 15 | 0.5 | 214 | 270 | 45 | 175 |
|  | 5983 | 38 | F | 7.4 | 157 | 14 | 0.5 | 173 | 247 | 40 | 135 |
|  | 39234 | 63 | M | >15 | 244 | 25 | 1.1 | 344 | 267 | 77 | 272 |
|  | 95924 | 74 | F | 8.8 | 105 | 19 | 0.7 | 190 | 168 | 47 | 149 |
|  | 5811 | 55 | F | 7.1 | 137 | 17 | 0.5 | 84 | 64 | 30 | 57 |
|  | 7396 | 63 | F | 6.1 | 112 | 20 | 0.7 | 206 | 190 | 46 | 159 |
|  | 106062 | 47 | F | 7.8 | 214 | 17 | 0.6 | 155 | 160 | 27 | 124 |
|  | 32761 | 54 | M | >15 | 296 | 26 | 0.9 | 240 | 334 | 57 | 156 |
|  | 32129 | 52 | M | 9.8 | 177 | 62 | 1.2 | 103 | 295 | 31 | 63 |
|  | 27019 | 38 | F | 7.8 | 154 | 13 | 0.6 | 160 | 95 | 65 | 100 |
|  | 105542 | 50 | F | 9.8 | 189 | 13 | 0.5 | 209 | 92 | 58 | 159 |
|  | 41488 | 52 | F | 6.7 | 108 | 22 | 0.7 | 185 | 158 | 39 | 146 |
|  | 1130 | 58 | F | 5.9 | 92 | 18 | 0.5 | 140 | 135 | 45 | 96 |
|  | 103955 | 65 | M | 11.3 | 220 | 35 | 0.8 | 173 | 124 | 36 | 138 |
|  | 32051 | 42 | F | 5.8 | 92 | 31 | 0.5 | 161 | 157 | 42 | 125 |
|  | 42665 | 42 | F | 6.7 | 130 | 17 | 0.6 | 123 | 350 | 32 | 75 |
|  | 5172 | 50 | M | 7.3 | 126 | 24 | 0.9 | 148 | 188 | 45 | 93 |
|  | 33997 | 59 | F | 7.6 | 177 | 28 | 0.9 | 228 | 153 | 67 | 149 |
|  | 54861 | 64 | F | 7.1 | 109 | 11 | 0.6 | 211 | 111 | 60 | 163 |
|  | 25048 | 56 | F | 5.7 | 106 | 16 | 0.6 | 186 | 122 | 51 | 141 |
|  | 4638 | 63 | M | 11.2 | 102 | 55 | 1.7 | 113 | 88 | 44 | 70 |
|  | 6150 | 50 | F | 9.8 | 132 | 24 | 0.6 | 192 | 163 | 44 | 42 |
|  | 5836 | 60 | F | >15 | 269 | 25 | 0.7 | 299 | 170 | 55 | 249 |
|  | 5854 | 47 | F | 9.7 | 189 | 19 | 0.8 | 168 | 237 | 42 | 124 |
|  | 5846 | 35 | M | >15 | 138 | 28 | 0.7 | 173 | 155 | 54 | 107 |
|  | 5892 | 52 | F | 11.5 | 231 | 12 | 0.6 | 151 | 232 | 39 | 115 |
|  | 5810 | 68 | M | 6.4 | 123 | 19 | 1.2 | 122 | 200 | 37 | 87 |
|  | 5833 | 53 | M | 8.6 | 197 | 19 | 0.8 | 134 | 300 | 28 | 102 |
|  | 35106 | 50 | F | 6.8 | 114 | 31 | 0.7 | 221 | 182 | 54 | 141 |
|  | 100525 | 44 | F | 10.5 | 84 | 30 | 1.1 | 257 | 262 | 54 | 197 |
|  | 38303 | 42 | M | 10.9 | 440 | 25 | 0.9 | 224 | 70 | 55 | 183 |
|  | 103482 | 48 | M | 5.6 | 91 | 25 | 0.9 | 147 | 71 | 57 | 94.7 |
|  | 99905 | 55 | F | 12.7 | 334 | 19 | 0.7 | 200 | 603 | 39 | 137.7 |
|  | 11340 | 59 | F | 5.8 | 109 | 20 | 0.6 | 156 | 93 | 55 | 106 |
|  | 10177 | 53 | F | 8.2 | 298 | 27 | 0.7 | 236 | 124 | 77 | 167 |
|  | 92300 | 59 | M | 6.2 | 149 | 68 | 1.8 | 137 | 74 | 54 | 93 |
|  | 37624 | 50 | F | 13.6 | 371 | 38 | 0.8 | 178 | 224 | 47 | 135 |
|  | 96353 | 70 | F | 9.2 | 171 | 15 | 0.7 | 176 | 235 | 36 | 138 |
|  | 104089 | 40 | F | 6.7 | 95 | 7 | 0.4 | 165 | 314 | 39 | 125 |
|  | 7708 | 52 | F | 8.9 | 269 | 22 | 0.6 | 200 | 313 | 63 | 138 |
|  | 100249 | 52 | F | 5.7 | 98 | 20 | 0.6 | 197 | 68 | 52 | 159 |
|  | 103966 | 38 | M | 8.8 | 102 | 29 | 0.9 | 123 | 102 | 37 | 94 |
|  | 99676 | 42 | M | 6.2 | 115 | 30 | 1.0 | 110 | 197 | 37 | 75 |
|  | 49904 | 69 | M | 7.2 | 160 | 28 | 0.9 | 148 | 89 | 42 | 114 |
|  | 1433 | 65 | M | 5.8 | 121 | 24 | 1.0 | 253 | 180 | 51 | 202 |
|  | 24663 | 33 | F | 10.9 | 114 | 15 | 0.7 | 174 | 85 | 63 | 125 |
|  | 35909 | 54 | F | 12.9 | 256 | 15 | 0.6 | 310 | 636 | 48 | 214 |
|  | 30906 | 57 | F | 5.7 | 152 | 22 | 0.8 | 105 | 79 | 42 | 64 |
|  | 26212 | 64 | M | 6.9 | 100 | 18 | 0.8 | 164 | 154 | 47 | 123 |
|  | 45331 | 62 | M | 5.8 | 77 | 44 | 0.7 | 96 | 61 | 45 | 59 |
|  | 46340 | 50 | M | 6.3 | 83 | 35 | 0.9 | 237 | 117 | 59 | 192 |
|  | 96593 | 39 | F | 5.3 | 87 | 16 | 0.7 | 229 | 145 | 53 | 178 |
|  | 5797 | 48 | F | 8.5 | 138 | 13 | 0.4 | 204 | 69 | 93 | 127 |
|  | 8211 | 65 | F | >15 | 184 | 37 | 0.8 | 218 | 283 | 38 | 140 |
|  | 43 | 63 | F | 8.9 | 161 | 22 | 0.7 | 153 | 114 | 44 | 114 |
|  | 12025 | 61 | F | 9.4 | 172 | 17 | 0.6 | 174 | 275 | 45 | 131 |
|  | 53457 | 58 | M | 5.9 | 126 | 40 | 0.9 | 123 | 92 | 36 | 92 |
|  | 37079 | 47 | F | 12.3 | 234 | 36 | 0.6 | 206 | 216 | 46 | 166 |
|  | 79795 | 75 | F | 9 | 170 | 34 | 0.8 | 110 | 94 | 32 | 82 |
|  | 32537 | 47 | M | 7.2 | 96 | 123 | 4.1 | 105 | 101 | 40 | 68 |
|  | 74359 | 59 | M | 6.6 | 140 | 20 | 0.9 | 146 | 81 | 39 | 97 |
|  | 7476 | 77 | F | 6.8 | 108 | 47 | 1.1 | 146 | 65 | 43 | 103 |
|  | 10752 | 43 | M | 5.7 | 94 | 21 | 0.8 | 142 | 93 | 45 | 103 |
|  | 20227 | 50 | F | 7 | 116 | 23 | 0.6 | 147 | 112 | 77 | 81 |
|  | 106496 | 62 | M | 11.4 | 218 | 49 | 1.2 | 104 | 131 | 34 | 74 |
|  | 105902 | 61 | F | 6.4 | 109 | 18 | 0.6 | 184 | 91 | 54 | 141 |
|  | 7273 | 43 | M | >15 | 222 | 17 | 1.0 | 144 | 83 | 69 | 88 |
|  | 34306 | 39 | M | 9.9 | 188 | 26 | 0.8 | 188 | 132 | 48 | 130 |
|  | 15791 | 66 | F | 11.8 | 156 | 23 | 1.2 | 111 | 106 | 30 | 80 |
|  | 59137 | 61 | M | 7.7 | 182 | 23 | 0.7 | 177 | 213 | 41 | 93 |
|  | 9291 | 41 | F | 6 | 96 | 15 | 0.8 | 173 | 99 | 44 | 130 |
|  | 26012 | 62 | M | 10.5 | 277 | 34 | 1.2 | 131 | 78 | 27 | 103 |
|  | 18563 | 37 | F | 6.4 | 87 | 13 | 0.7 | 140 | 66 | 46 | 99.5 |
|  | 21720 | 54 | F | >15 | 472 | 27 | 1.3 | 252 | 221 | 62 | 189 |
|  | 33580 | 62 | F | 9.1 | 266 | 14 | 0.9 | 173 | 108 | 54 | 123 |
|  | 48766 | 42 | F | 9.1 | 87 | 27 | 0.9 | 214 | 63 | 64 | 161 |
|  | 87949 | 67 | M | 8.9 | 112 | 26 | 1.3 | 155 | 65 | 46 | 116 |
|  | 8211 | 65 | F | >15 | 184 | 37 | 0.8 | 218 | 283 | 38 | 140 |
|  | 26363 | 40 | F | 6.7 | 117 | 21 | 0.7 | 135 | 96 | 31 | 97 |
|  | 65099 | 23 | M | >15 | 358 | 12 | 0.6 | 129 | 71 | 43 | 91 |
|  | 107247 | 45 | M | 5.8 | 103 | 15 | 0.6 | 203 | 262 | 35 | 179 |
|  | 68679 | 39 | F | 5.4 | 101 | 12 | 0.7 | 122 | 140 | 43 | 78 |
|  | 32094 | 74 | F | 8.3 | 185 | 50 | 1.8 | 176 | 119 | 47 | 128 |
|  | 106784 | 52 | M | 9.5 | 243 | 31 | 1.5 | 188 | 219 | 44 | 138 |
|  | 39511 | 64 | F | 8.8 | 177 | 14 | 0.4 | 105 | 83 | 39 | 68 |
|  | 7474 | 49 | M | 5.7 | 110 | 30 | 1.2 | 159 | 116 | 44 | 120 |
|  | 106869 | 58 | M | 5.8 | 101 | 46 | 1.2 | 89 | 50 | 24 | 61.1 |
|  | 25655 | 70 | M | 9.6 | 131 | 12 | 0.7 | 164 | 81 | 39 | 126 |
|  | 58809 | 51 | M | 7.2 | 90 | 25 | 0.8 | 176 | 170 | 41 | 131 |
|  | 53394 | 61 | F | 8.7 | 166 | 19 | 0.6 | 135 | 121 | 42 | 97 |
|  | 29725 | 52 | M | 6.6 | 90 | 17 | 1.0 | 157 | 97 | 41 | 118.4 |
|  | 13068 | 77 | M | 8.6 | 144 | 40 | 1.2 | 147 | 120 | 52 | 104 |
|  | 12785 | 53 | M | 11.5 | 116 | 18 | 1.0 | 124 | 88 | 42 | 86.5 |
|  | 24101 | 50 | M | 5.7 | 107 | 21 | 0.7 | 182 | 84 | 47 | 142 |
|  | 30107 | 65 | M | 9.6 | 324 | 90 | 4.2 | 196 | 340 | 34 | 152 |
|  | 32085 | 41 | F | 8.6 | 100 | 13 | 0.7 | 125 | 72 | 34 | 93 |
|  | 2663 | 67 | F | 12.1 | 90 | 24 | 0.6 | 120 | 113 | 62 | 67.3 |
|  | 2803 | 37 | F | >15 | 342 | 12 | 0.6 | 209 | 140 | 45 | 173.2 |
|  | 16397 | 52 | F | 5.8 | 98 | 20 | 0.8 | 137 | 46 | 57 | 89 |
|  | 107570 | 75 | M | 5.6 | 99 | 17 | 0.6 | 178 | 113 | 45 | 136.5 |
|  | 64763 | 58 | F | 11.8 | 267 | 16 | 0.5 | 182 | 177 | 38 | 144 |
|  | 58980 | 49 | M | 9.6 | 172 | 43 | 1.3 | 219 | 114 | 60 | 171 |
|  | 38135 | 54 | M | 14.4 | 287 | 17 | 0.7 | 93 | 77 | 26 | 69 |
|  | 4870 | 50 | M | 7.9 | 88 | 23 | 1.2 | 125 | 142 | 44 | 84 |
|  | 107107 | 54 | M | 5.8 | 99 | 100 | 5.8 | 151 | 154 | 43 | 116 |
|  | 102529 | 35 | F | 7.3 | 170 | 31 | 0.8 | 304 | 361 | 64 | 228 |
|  | 107440 | 57 | F | 8.5 | 140 | 21 | 0.6 | 153 | 138 | 45 | 105.6 |
|  | 109416 | 40 | M | 6.3 | 84 | 21 | 1.0 | 117 | 120 | 33 | 86.2 |
|  | 16615 | 76 | M | 9.2 | 133 | 29 | 1.2 | 156 | 80 | 50 | 115 |
|  | 32165 | 59 | F | 6.6 | 90 | 22 | 0.6 | 122 | 73 | 48 | 83 |
|  | 96762 | 54 | M | 10.4 | 150 | 14 | 0.7 | 309 | 146 | 66 | 247 |
|  | 60210 | 38 | F | 6.2 | 87 | 13 | 0.8 | 130 | 75 | 26 | 110 |
|  | 16717 | 48 | F | 6.6 | 173 | 20 | 0.6 | 150 | 116 | 55 | 104 |
|  | 91642 | 51 | F | 8.8 | 162 | 9 | 0.5 | 152 | 86 | 40 | 112 |
|  | 81719 | 67 | M | 7.3 | 130 | 28 | 1.0 | 187 | 142 | 38 | 126 |
|  | 37646 | 47 | F | 10.6 | 151 | 20 | 0.6 | 188 | 88 | 65 | 132 |
|  | 16275 | 60 | M | 6.8 | 94 | 12 | 0.8 | 211 | 310 | 40 | 168 |
|  | 27733 | 59 | F | 9.6 | 154 | 19 | 0.8 | 134 | 90 | 41 | 98 |
|  | 31099 | 49 | F | 10 | 191 | 26 | 0.9 | 141 | 151 | 40 | 101 |
|  | 24196 | 50 | F | 6.9 | 78 | 25 | 0.8 | 181 | 129 | 52 | 128 |
|  | 26098 | 42 | M | 11.3 | 168 | 10 | 0.8 | 118 | 56 | 42 | 85.7 |
|  | 28413 | 61 | F | 8.6 | 133 | 15 | 0.7 | 138 | 164 | 38 | 102.4 |
|  | 37916 | 37 | F | 7 | 96 | 22 | 0.9 | 195 | 166 | 56 | 147.8 |
|  | 45961 | 29 | F | 10.9 | 205 | 16 | 0.7 | 150 | 101 | 47 | 105 |
|  | 31288 | 42 | F | 7.5 | 211 | 13 | 0.5 | 173 | 146 | 38 | 148.6 |
|  | 67024 | 62 | M | 6.8 | 116 | 16 | 0.9 | 99 | 112 | 32 | 75 |
|  | 3233 | 52 | F | 8.2 | 153 | 12 | 0.5 | 125 | 86 | 60 | 79 |
|  | 68934 | 49 | M | 9.4 | 240 | 115 | 1.3 | 134 | 109 | 35 | 109 |
|  | 107768 | 56 | M | 10.7 | 108 | 19 | 0.8 | 178 | 141 | 51 | 128 |
|  | 17702 | 39 | M | 6.7 | 112 | 45 | 0.7 | 139 | 58 | 58 | 90 |
|  | 24806 | 55 | F | 8.2 | 112 | 88 | 1.5 | 113 | 98 | 50 | 74 |
|  | 31523 | 62 | M | 6.7 | 143 | 46 | 1.1 | 137 | 191 | 41 | 108 |
|  | 107600 | 40 | F | 10.5 | 325 | 29 | 0.5 | 93 | 111 | 36 | 67 |
|  | 32899 | 67 | M | 5.1 | 106 | 22 | 0.8 | 152 | 80 | 50 | 103 |
|  | 9932 | 41 | M | 5 | 82 | 34 | 0.5 | 170 | 72 | 51 | 137 |
|  | 20755 | 60 | F | 7.5 | 102 | 12 | 0.5 | 123 | 109 | 55 | 81 |
|  | 107291 | 32 | F | 5.3 | 83 | 10 | 0.5 | 135 | 55 | 39 | 103 |
|  | 107247 | 45 | M | 5.8 | 103 | 15 | 0.6 | 203 | 262 | 35 | 179 |
|  | 6591 | 31 | F | 5.9 | 115 | 20 | 0.6 | 153 | 68 | 56 | 104 |
|  | 30509 | 41 | F | 9.3 | 164 | 20 | 0.4 | 174 | 145 | 35 | 155 |
|  | 12436 | 43 | F | 7.2 | 151 | 15 | 0.6 | 141 | 153 | 53 | 93 |
|  | 24064 | 79 | M | 7.5 | 191 | 17 | 0.5 | 175 | 129 | 41 | 151 |
|  | 26152 | 52 | M | 9.1 | 181 | 45 | 1.3 | 90 | 142 | 22 | 61 |
|  | 46290 | 57 | M | 9.3 | 146 | 33 | 0.9 | 110 | 48 | 42 | 69 |
|  | 24378 | 62 | M | 8.3 | 116 | 14 | 0.8 | 122 | 94 | 39 | 95 |
|  | 11256 | 68 | M | 7.9 | 175 | 89 | 4.3 | 198 | 175 | 69 | 141 |
|  | 20242 | 47 | M | 6.8 | 156 | 21 | 0.7 | 157 | 217 | 47 | 120 |
|  | 94318 | 45 | F | >15 | 207 | 18 | 0.5 | 174 | 167 | 41 | 110 |
|  | 34995 | 62 | M | 10.5 | 137 | 18 | 1.0 | 108 | 91 | 46 | 74 |
|  | 107849 | 27 | F | 6.6 | 92 | 19 | 0.5 | 164 | 207 | 25 | 147 |
|  | 95647 | 25 | F | 11.3 | 311 | 16 | 0.6 | 144 | 49 | 67 | 94.8 |
|  | 39436 | 52 | F | 7.6 | 128 | 17 | 0.4 | 154 | 72 | 61 | 112 |
|  | 32073 | 21 | F | 7.9 | 243 | 15 | 0.6 | 101 | 51 | 43 | 71 |
|  | 97922 | 38 | F | 5 | 92 | 21 | 0.5 | 112 | 47 | 56 | 67 |
|  | 36399 | 72 | M | 8.8 | 252 | 23 | 0.9 | 112 | 74 | 39 | 87 |
|  | 25938 | 62 | F | 9 | 192 | 12 | 0.5 | 156 | 205 | 38 | 128 |
|  | 38331 | 47 | M | 6.8 | 145 | 14 | 0.8 | 177 | 381 | 39 | 126 |
|  | 65151 | 57 | F | 8.5 | 142 | 33 | 0.7 | 126 | 73 | 53 | 89 |
|  | 104034 | 35 | M | 5.4 | 92 | 11 | 0.8 | 174 | 244 | 36 | 136 |
|  | 102648 | 48 | F | 6.9 | 107 | 16 | 0.6 | 233 | 164 | 52 | 202 |
|  | 33408 | 37 | F | 10 | 218 | 12 | 0.5 | 168 | 125 | 49 | 136 |
|  | 94318 | 45 | F | >15 | 207 | 18 | 0.5 | 174 | 167 | 41 | 110 |
|  | 44426 | 67 | M | 10 | 206 | 97 | 2.6 | 137 | 184 | 35 | 102 |
|  | 31899 | 43 | F | 9.5 | 104 | 13 | 0.6 | 213 | 120 | 53 | 180 |
|  | 68769 | 71 | M | 8.4 | 116 | 55 | 1.7 | 112 | 249 | 23 | 83.7 |
|  | 7072 | 72 | F | 5.6 | 118 | 11 | 0.7 | 189 | 75 | 46 | 122 |
|  | 29967 | 48 | M | 6.1 | 103 | 32 | 0.9 | 198 | 241 | 44 | 154 |
|  | 5097 | 53 | F | 5.4 | 101 | 17 | 0.5 | 152 | 115 | 46 | 115 |
|  | 26328 | 45 | M | 8.7 | 210 | 27 | 0.8 | 150 | 105 | 46 | 114 |
|  | 24846 | 30 | F | 6.9 | 291 | 15 | 0.5 | 125 | 49 | 48 | 86.4 |
|  | 105300 | 38 | M | 8.5 | 149 | 11 | 0.7 | 220 | 214 | 43 | 178 |
|  | 14133 | 35 | F | 9.1 | 217 | 18 | 0.6 | 149 | 145 | 42 | 110 |
|  | 3174 | 53 | F | 7.5 | 132 | 7 | 0.6 | 176 | 107 | 54 | 131 |
|  | 27238 | 55 | F | 6.8 | 103 | 18 | 0.6 | 163 | 155 | 48 | 99 |
|  | 108311 | 40 | M | 8.4 | 222 | 20 | 0.8 | 169 | 143 | 32 | 144 |
|  | 191229 | 57 | F | 9.6 | 210 | 23 | 0.5 | 252 | 109 | 52 | 157 |
|  | 192907 | 39 | F | 11.6 | 237 | 19 | 0.5 | 245 | 129 | 56 | 201 |
|  | 192856 | 73 | M | 5.9 | 90 | 16 | 0.7 | 181 | 85 | 66 | 118 |
|  | 192879 | 38 | F | 7.6 | 137 | 32 | 1.8 | 266 | 322 | 58 | 194 |
|  | 191206 | 39 | F | 9.6 | 230 | 13 | 0.6 | 116 | 66 | 57 | 61 |
|  | 193025 | 58 | M | 8.2 | 98 | 96 | 2.6 | 91 | 123 | 31 | 60 |
|  | 193073 | 44 | F | 10.5 | 257 | 23 | 0.8 | 190 | 126 | 63 | 133 |
|  | 193783 | 75 | M | 8.3 | 260 | 28 | 1.0 | 99 | 99 | 39 | 62 |
|  | 193660 | 66 | M | 7.2 | 145 | 16 | 0.8 | 115 | 158 | 44 | 68 |
|  | 193747 | 65 | M | 6.1 | 108 | 21 | 0.9 | 161 | 347 | 42 | 114 |
|  | 193636 | 72 | F | 10.1 | 191 | 15 | 0.7 | 115 | 104 | 37 | 80 |
|  | 195207 | 42 | M | 10.3 | 208 | 20 | 0.7 | 170 | 131 | 48 | 121 |
|  | 195359 | 57 | M | 9.3 | 246 | 31 | 1.0 | 93 | 149 | 38 | 53 |
|  | 195421 | 50 | M | 9.8 | 165 | 14 | 0.8 | 211 | 383 | 49 | 149.7 |
|  | 198174 | 49 | F | 9.5 | 316 | 20 | 0.7 | 163 | 113 | 63 | 108 |
|  | 198465 | 38 | F | 9.3 | 209 | 22 | 0.4 | 168 | 244 | 51 | 120 |
